# Epigallocatechin Gallate in Relapsing-Remitting Multiple Sclerosis: A Randomized, Placebo-Controlled Trial

**DOI:** 10.1212/NXI.0000000000000981

**Published:** 2021-03-24

**Authors:** Judith Bellmann-Strobl, Friedemann Paul, Jens Wuerfel, Jan Dörr, Carmen Infante-Duarte, Elmira Heidrich, Benedict Körtgen, Alexander Brandt, Caspar Pfüller, Helena Radbruch, Rebekka Rust, Volker Siffrin, Orhan Aktas, Christoph Heesen, Jürgen Faiss, Frank Hoffmann, Mario Lorenz, Benno Zimmermann, Sergiu Groppa, Klaus-Dieter Wernecke, Frauke Zipp

**Affiliations:** From the NeuroCure Clinical Research Center (J.B.-S., F.P., J.D., A.B., V.S.), Charité—Universitätsmedizin Berlin; Medical Image Analysis Center (J.W.), University Basel; Institut for Medical Immunology (C.I.-D., E.H.), Charité—Universitätsmedizin Berlin; Department of Neurology and Neuroimaging Center (B.K.), Johannes Gutenberg University, Mainz; Charité—Universitätsmedizin Berlin (C.P.); NeuroCure Clinical Research Center (H.R., R.R.), Charité—Universitätsmedizin Berlin, Germany; Department of Neurology (O.A.), Medical Faculty, Heinrich Heine University Düsseldorf; Institut für Neuroimmunologie und Multiple Sklerose (C.H.), Universitätsklinikum Hamburg-Eppendorf, Hamburg; Klinik für Neurologie (J.F.), Asklepios Klinik Lübben/Teupitz; Department of Neurology (F.H.), Krankenhaus Martha-Maria Halle-Dölau, Halle/Saale; Medizinische Klinik für Kardiologie und Angiologie (M.L.), Campus Mitte, Charité—Universitätsmedizin Berlin; Institute of Nutritional and Food Sciences (B.Z.), University of Bonn; Department of Neurology and Neuroimaging Center (NIC) (S.G., F.Z.), Focus Program Translational Neuroscience (FTN), University Medical Center of the Johannes Gutenberg University, Mainz; and Charité—Universitätsmedizin Berlin and SOSTANA GmbH (K.-D.W.), Berlin.

## Abstract

**Objective:**

To assess the safety and efficacy of epigallocatechin-3-gallate (EGCG) add-on to glatiramer acetate (GA) in patients with relapsing-remitting multiple sclerosis (RRMS).

**Methods:**

We enrolled patients with RRMS (aged 18–60 years, Expanded Disability Status Scale [EDSS] score 0–6.5), receiving stable GA treatment in a multicenter, prospective, double-blind, phase II, randomized controlled trial. Participants received up to 800 mg oral EGCG daily over a period of 18 months. The primary outcome was the proportion of patients without new hyperintense lesions on T2-weighted (T2w) brain MRI within 18 months. Secondary end points included additional MRI and clinical parameters. Immunologic effects of EGCG were investigated in exploratory experiments.

**Results:**

A total of 122 patients on GA were randomly assigned to EGCG treatment (n = 62) or placebo (n = 60). We could not demonstrate a difference between groups after 18 months for the primary outcome or other radiologic (T2w lesion volume, T1w hypointense lesion number or volume, number of cumulative contrast-enhancing lesions, percent brain volume change), or clinical (EDSS, MS functional composite, and annualized relapse rate) parameter. EGCG treatment did not affect immune response to GA. Pharmacologic analysis revealed wide ranging EGCG plasma levels. The treatment was well tolerated with a similar incidence of mostly mild adverse events similar in both groups.

**Conclusion:**

In RRMS, oral EGCG add-on to GA was not superior to placebo in influencing MRI and clinical disease activity over 18 months. The treatment was safe at a daily dosage up to 800 mg EGCG. It did not influence immune parameters, despite indication of EGCG being bioavailable in patients.

**Classification of Evidence:**

This study provides Class II evidence that for patients with RRMS, EGCG added to GA did not significantly affect the development of new hyperintense lesions on T2-weighted brain MRI.

**Trial Registration Information:**

Clinical trial registration number: NCT00525668.

Multiple sclerosis (MS) is characterized by autoimmune inflammatory and neurodegenerative pathology of the CNS causing pronounced neurologic disability in younger adults.^1,2^ In recent years, several immunomodulatory drugs for the treatment of relapsing-remitting MS (RRMS) have been approved targeting mainly the inflammatory processes of this disease.^3,4^

However, the development of drugs that are capable of halting or decelerating the neurodegenerative aspects, which are prevalent from the earliest disease stages, is an unmet clinical need.^5^

Consumption of green tea is considered to have a preventive impact on various inflammatory and neurodegenerative as well as other diseases.^6,7^ The most relevant compound in this regard is the polyphenol epigallocatechin gallate (EGCG), comprising 50–80% of the total catechins in green tea.^8^

In experimental autoimmune encephalomyelitis (EAE)—an animal model mimicking aspects of MS—EGCG exerts anti-inflammatory properties via downregulation of NF-κB in T cells and has neuroprotective capacities by blocking the formation of neurotoxic reactive oxygen species in neurons.^9^ In this model, oral EGCG significantly reduced clinical disease severity as well as CNS inflammation and neuroaxonal damage, both as preventive and therapeutic treatment.^9,10^ Moreover, in EAE, concomitant application of EGCG and glatiramer acetate (GA) revealed synergistic effects in vitro and in vivo.^11^

Against this background, we investigated the effect of oral EGCG given as add-on to GA therapy over a period of 18 months on radiologic and clinical disease activity as well as safety and tolerability in patients with RRMS.

## Methods

### Primary Research Question

We performed a prospective, double-blind, parallel-group, randomized controlled trial in patients with RRMS, at 9 sites in Germany (including general hospitals and academic medical centers) recruiting from August 2007 to May 2011 to evaluate the question whether oral application of up to 800 mg EGCG reduces the development of new hyperintense lesions on T2-weighted (T2w) brain MRI in patients with RRMS on stable treatment with GA 20 mg. This study provides Class II evidence because less than 80% of randomized patients completed the trial.

### Study Design and Participants

For details on the study conduct, refer to the study protocol in the online supplement. Eligibility criteria comprised fulfillment of the 2005 McDonald criteria for RRMS,^12^ age between 18 and 60 years, an Expanded Disability Status Scale (EDSS)^13^ score of 0–6.5, and a stable treatment with GA 20 mg daily subcutaneously for at least 6 months. A relapse-free period of at least 30 days before randomization was mandatory. Key exclusion criteria were any progressive forms of MS, major systemic disease, clinically relevant predefined laboratory abnormalities, and intake of any potentially hepatotoxic medication as well as cytochrome P450 3A4–inhibiting or –inducing drugs. Additional consumption of green tea or GTE was prohibited.

Because of lacking human data, sample size calculation was based on articles by Aktas et al.^9^ and Zhao et al.^14^ Proportions of 45% for EGCG and 16% for placebo were assumed for the primary end point (patients without new T2w lesions after 18 months), leading to 92 patients in total (2-sided type 1 error = 5%, power = 80%). Because of uncertainty in the preconditions of the sample size calculation, an internal pilot study^15^ was integrated into the study. This design allows for a (blinded) recalculation of sample size without affecting the type I error.^16^ The planned recalculation after the inclusion of 50 participants resulted in a sample size of 126 patients in total, assuming a prior difference of 0.20 between proportions. This sample size was confirmed by a second internal blinded recalculation after inclusion of 80 individuals.

### Standard Protocol Approvals, Registrations, and Patient Consents

The study was approved by the local ethics committees and by the German Federal Institute for Drugs and Medical Devices (BfArM). This trial is registered with EudraCT (Nr. 2006-006323-39) and clinicaltrials.gov (NCT00525668). It was conducted strictly following the study protocol, the applicable German laws (Arzneimittelgesetz, 14. Novelle 2005), the Harmonized Tripartite Guideline for Good Clinical Practise (ICH-GCP), and the principles of the Declaration of Helsinki in its applicable version. Every participant provided written informed consent before enrollment.

### Data Availability Statement

As far as permitted according to data protection requirements and consent provided by the participants, original data are available from the corresponding author on request from any qualified investigator within 5 years after publication.

### Randomization and Masking

Patients were randomly (1:1) assigned to receive as add-on to GA after a dosing phase of 4 months per day either 800 mg capsules of Sunphenon® (GTE containing >90% EGCG, product of Taiyo International, taiyointernational.com) or capsules of placebo, which had identical appearance.

To account for potential baseline imbalances, patients were stratified before randomization for sex (female/male) and T2w lesion number at screening (≤15 or >15 T2w lesions). A separate block randomization list was generated by the independent pharmacy, which distributed the screened study participants to the treatment groups.

Patients and all staff remained masked for treatment allocation during the entire study. To minimize the risk of biased clinical examinations by patients reporting adverse events (AE), an independent examining physician restricted to performing the neurologic examination rated EDSS only.

### Procedures

Standardized neurologic assessments including the EDSS^13^ and Multiple Sclerosis Functional Composite (MSFC)^17^ with its subtests 9-Hole Peg Test, Timed 25-Foot Walk Test, and Paced Auditory Serial Addition Test (PASAT) were performed by an especially trained and neurostatus-certified examiner at screening (which was at most 1 week before randomization), then every 3 months until the end of the study at month 18, and at every unscheduled visit when a relapse was suspected. A relapse was defined as any new or reoccurring neurologic symptoms in the absence of fever or infections, lasting for at least 24 hours, separated by at least 30 days from the onset of a previous relapse, and confirmed by the independent EDSS rater. For safety monitoring, regular medical examinations und laboratory examinations (blood count, liver enzymes, electrolytes, creatinine, C-reactive protein, blood glucose, and coagulation) were scheduled every 3 months and in short-term follow-up in case of pathologic results.

MRI was performed at screening and thereafter every 3 months until the end of the study at month 18. For all study sites, MRI measurements were performed at a single central facility (leading study site Charité) ensuring identical and constant acquisition conditions on a 1.5 T MRI (Siemens Sonata, Siemens Medical Systems, Erlangen, Germany).

To investigate potential immunologic effects of EGCG treatment, we analyzed the frequencies and activation status of T cells (CD4^+^ and CD8^+^), B cells, monocytes, and natural killer (NK) cells by flow cytometry analysis using EDTA whole blood samples from a randomly selected subgroup of 35 study participants (20 EGCG and 15 placebo). Furthermore, to assess the specific proliferative response to GA, peripheral blood mononuclear cells (PBMCs) were isolated from patients' whole blood (n = 40 EGCG group; n = 39 placebo group including the 35 patients of the immunologic substudy).

To measure EGCG plasma levels, biosamples were acquired at a time point after overnight fasting and before intake of the first dose of study medication (200 mg EGCG or placebo capsule) as well as 2 hours later after a standardized breakfast. Plasma concentrations of EGCG were determined as previously described.^18^

### Outcomes

The primary outcome was the proportion of patients without new hyperintense T2w MRI lesions within 18 months. Secondary MRI outcomes were number and volume of T2w hyperintense lesions, number and volume of T1w hypointense lesions (black holes), number of cumulative contrast-enhancing lesions (CELs), and brain atrophy quantified by percent brain volume change (PBVC). Secondary clinical outcome measurements were disability progression measured by EDSS and MSFC as well as annualized relapse rate. Immunologic effects of EGCG were assessed in exploratory experiments.

### Statistical Analysis

An intention-to-treat (ITT) approach was planned as the primary analysis. In addition, a per-protocol (PP) analysis was performed, omitting patients with major protocol violations, i.e., who stopped treatment due to adverse reaction or who disregarded study procedures, defined as missing more than 2 scheduled study visits or intake of less than 90% of the study medication.

Results are expressed as arithmetic mean ± SD, median (range), or frequencies (%). The primary end point was assessed using the Fisher exact test. Secondary end points were tested for differences between groups by using the nonparametric (exact) Mann-Whitney test for independent groups. Differences in categorical variables were tested by the Fisher exact test.

Differences between the EGCG and placebo groups for the entire time course were assessed using nonparametric multivariate variance and covariance analyses of all longitudinal data in a two-factorial design.^19^ A *p* value <0.05 was considered statistically significant. All tests of secondary end points were conducted as exploratory data analysis. Therefore, no adjustments for multiple testing were made. Numerical calculations were performed with IBM SPSS Statistics for Windows, version 21 (IBM Corp., Armonk, NY), StatXact 6 (CYTEL Software Corp., Cambridge, MA), and SAS, version 9.2 (SAS Institute, Inc., Cary, NC).

## Results

**Figure 1:**
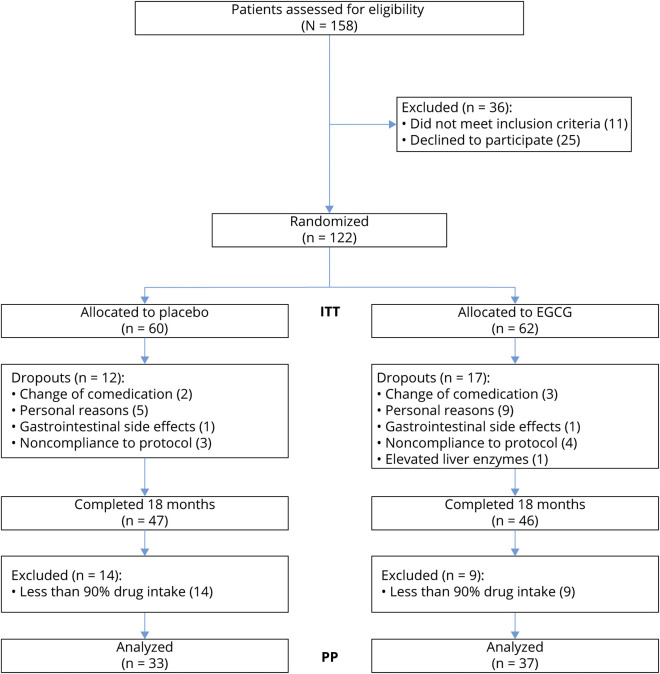
Trial Flowchart ITT = intention to treat; PP = per protocol.

One hundred fifty-eight patients were screened for eligibility, 122 of whom were enrolled in the study (figure 1). Participants were randomly assigned to receive EGCG (n = 62) or placebo (n = 60) as add-on to immunomodulatory therapy with GA. All included patients were of Caucasian ethnicity. The 2 groups did not differ regarding baseline variables (table 1).

**Table 1:**
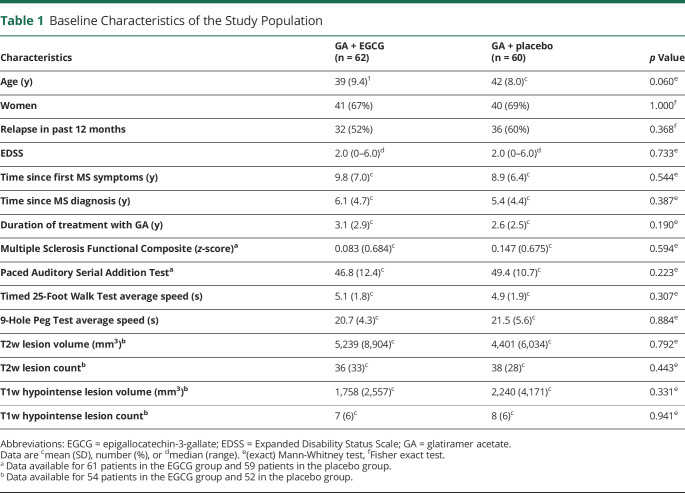
Baseline Characteristics of the Study Population

Ultimately, 17 patients in the EGCG group and 12 patients in the placebo group did not complete the study (figure 1). This was mainly due to personal reasons (such as relocation or the desire to become pregnant), change from GA to other disease-modifying therapy, or noncompliance of study rules (e.g., missing more than 2 visits or breaking the blinding by having the study medication analyzed by a third party). One patient in each group discontinued due to stomach and digestion complaints. In the EGCG group, 1 participant had to stop study medication because of elevated liver enzymes higher than threefold the upper limit of normal; elevated values normalized thereafter. Of the patients completing the full 18 months of study medication, 33 patients (68.8%) on placebo and 37 patients (82.2%) on EGCG had a compliance of at least 90% regarding intake of study medication (number of taken capsules as assessed by the drug count at study visits).

**Figure 2:**
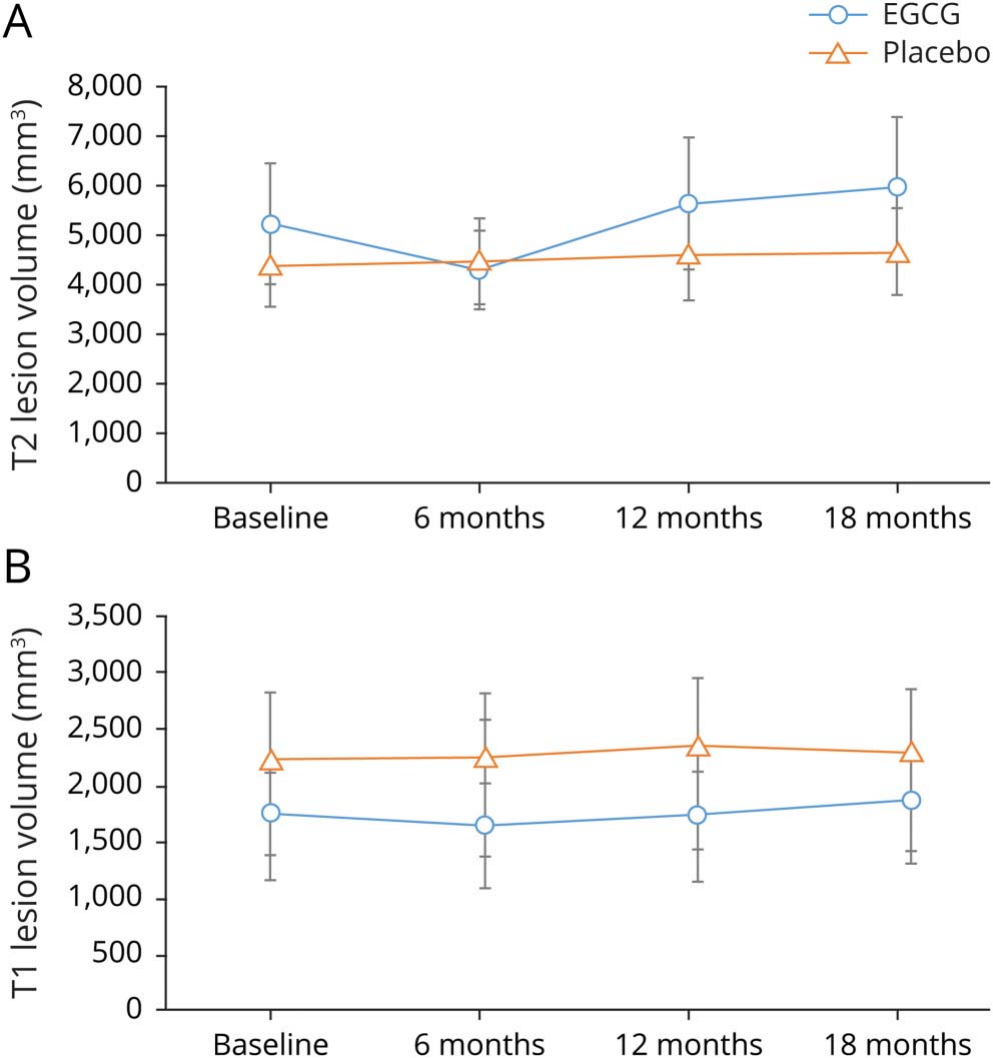
T2w and T1w Hypointense Lesion Volumes Mean change over time of volume (A) T2w lesion load (B) T1w hypointense lesion. EGCG = epigallocatechin-3-gallate.

The results of the ITT analyses for the MRI outcome parameters are summarized in table 2. Regarding the primary end point, we observed no significant difference in the proportion of patients without new T2w hyperintense lesions between EGCG- and placebo-treated patients at month 18. Regarding secondary outcomes, the number of T2w lesions as well as the number of T1w hypointense lesions (table 2) increased irrespective of EGCG or placebo group during the study period as did the volume of T2w hyperintense and T1w hypointense lesions (figure 2 and table 2). Neither parameter revealed significant differences between the study groups (table 2).

**Table 2:**
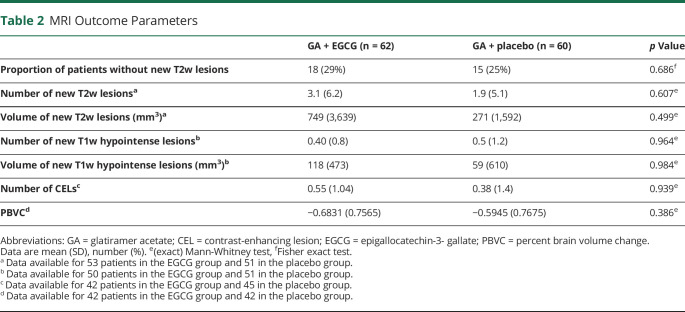
MRI Outcome Parameters

Longitudinal analysis of the entire time course^19^ including all available time points (0, 6, 12, 15, and 18 months) adjusted for values at baseline also did not show a significant difference in MRI parameters between the EGCG and placebo groups (data not shown). Both groups developed a similar number of CELs during the study.

Furthermore, we could not detect a difference between the 2 study groups in PBVC, a measure of whole-brain atrophy, over the 18-month period of the trial (table 2).

With regard to clinical end points (table 3), no differences were observed in EDSS or MSFC between the EGCG and placebo groups, neither in regard to change from baseline to month 18 nor in longitudinal EDSS analysis of the entire study course adjusted for values at baseline.

**Table 3:**
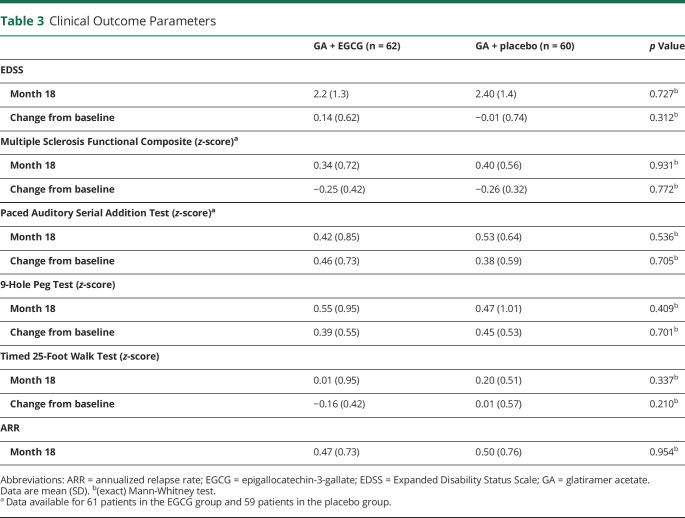
Clinical Outcome Parameters

The results of the PP analyses (n = 70) concerning primary as well as all secondary outcome parameters did not differ in their statistical significance from those of the ITT analyses (data not shown).

The analysis of subgroups enables a differentiated view. In the group of participants who did not suffer a relapse 12 months before study inclusion, the rate of patients who did not develop new T2w lesions was higher in the group with the active ingredient (EGCG 12/21, placebo 5/19, p = 0.062). This was also the case in the group of participants with 15 and lower T2w lesions at baseline (EGCG 10/13, placebo 3/9, p = 0.079) and surprisingly in the subgroup of patients who developed T2w lesion volume increase during the study (EGCG 10/38, placebo 3/35, *p* = 0.067). No statistically significant difference in the rate of newly developed T2w lesions could be demonstrated in the subgroup with EDSS 3 and lower (EGCG 13/36, placebo 11/39, *p* = 0.621) and in the subgroup of patients with Gd-positive lesions in the course of the study (EGCG 3/19, placebo 3/11, *p* = 0.641).

The analysis of the frequencies of the main immune cell populations (numbers of circulating T cells, B cells, monocytes, or NK cells) in a subgroup of 20 EGCG-treated and 15 placebo-treated patients revealed that the treatment with EGCG did not alter the overall immune status of the patients (data not shown). The in vitro examination of the T-cell response to different concentrations of GA using PBMC from 40 EGCG and 39 placebo-treated patients indicated that treatment with EGCG did not interfere with the overall T-cell response of the patients to GA. Though, EGCG treatment had a tendency to diminish the in vitro response to high GA concentrations at 2 mg/mL (*p* = 0.099, data not shown). This concentration is far higher than the serum level in humans under regular treatment with GA.

Of the 60 participants in the placebo group, 58 (97%) experienced 1 or more AEs, with 8 (13%) having a serious adverse event (SAE). In the EGCG group, 60 of the 62 participants (97%) had at least 1 AE, 6 (10%) of which were considered serious (see table e-1, links.lww.com/NXI/A458). None of the SAEs were considered related to the study drug. All occurred due to hospitalization of study participants for various reasons. The incidence of SAE and AE was similar in both study groups. The most common AEs were infections of the upper respiratory, gastrointestinal, and urinary tracts. One placebo-treated and 1 EGCG-treated patient stopped intake of study medication because of gastrointestinal complaints. As 1 patient of the EGCG group had to be withdrawn due to elevated liver enzymes, we performed a comparison of liver enzyme levels between our study groups. This revealed no significant differences (data not shown). In plasma from patients on placebo, EGCG was not detectable at any time point.

In 41 patients on EGCG, 2 hours after ingestion of a standardized breakfast and the morning dose of 200 mg Sunphenon, EGCG plasma levels ranging from 20.21 to 331.66 ng/mL were measured. If the data set is divided into 2 groups at the median of the EGCG level, the number of new T2w lesions is less in the group with higher EGCG levels compared with the group below the median. But the numbers are too small for statistical significance.

## Discussion

Our randomized, placebo-controlled multicenter study failed to show an effect of oral EGCG on MRI or clinical disease markers in patients with RRMS on stable immunomodulatory treatment with GA.

In line with other polyphenols, experimental data had previously demonstrated that orally administered EGCG reduced disease severity when given at initiation or after the onset of experimental neuroinflammation and exerted both anti-inflammatory and neuroprotective effects, also in combination with GA.^9,11^ Among the potential mechanisms of action of EGCG are antioxidant properties and an impact on several signal transduction pathways, including growth factor–mediated pathways, the mitogen-activated protein kinase–dependent, and ubiquitin/proteasome degradation pathways. These data in conjunction with EGCG's presumed mode of action together with the finding that the conventional form of MS typical for Western countries is much less prevalent in Asian countries with high green tea consumption like Japan^20^ encouraged us to conduct this randomized placebo-controlled add-on trial.

Only a few clinical studies with patients with cancer administering high-dose EGCG or GTE had been reported before planning of our trial.^21,22^ For the selection of the maximum daily dose of EGCG, we had to rely on pharmacokinetic and tolerability studies in healthy subjects with short-time intakes only (maximum weeks) of doses from 800 to 1,000 mg EGCG/GTE per day.^23–25^ Plasma elimination half-life of EGCG was reported to be as long as 5.2 hours, and levels were detectable after repeated administration of 800 mg EGCG once daily over 10 days.^25^ We therefore concluded that 400 mg EGCG twice daily would be sufficient to yield measurable plasma levels even after overnight fasting. However, we have learned from our data that plasma levels of EGCG are extremely variable between individuals even under standardized conditions. Although a dosage of 600 mg EGCG daily improved muscle metabolism in patients with MS,^26^ oral ingestion of 400 mg EGCG twice daily may not have been sufficient to exert biological effects in the CNS in all patients due to insufficient plasma levels. This may be 1 potential explanation for the negative outcome of this study. Recently, the bioavailability of orally administered EGCG was called into question,^27^ which is however in contradiction to an earlier pharmacokinetic study reporting a high absorption rate of oral EGCG in the fasting state.^25^

Another putative cause for not meeting the efficacy end points could lie in the add-on study design. A placebo-controlled EGCG-only trial would have been considered unethical and would not be approved by the competent authorities. Thus, we had to choose an add-on approach to an approved immunomodulatory drug. For reasons of safety, we selected GA because we considered this compound to be the least problematic in terms of potential unfavorable drug interactions, in particular as hepatotoxicity had been discussed as a rare but potentially severe side effect of green tea dietary supplements.^28^ Fortunately, we did not face SAEs with our EGCG dosing regimen and GA combination therapy. This is in contrast to a small study with Polyphenon E (a GTE compound) in MS that was prematurely terminated due to significant hepatotoxicity.^29^ In our study, only 1 subject dropped out due to elevated liver enzymes. Maybe EGCG as a pure substance afflicts metabolic processes of the liver less than GTE, containing several types of polyphenols and sometimes small amounts of caffeine in addition.

Furthermore, in the study with Polyphenon E, add-on therapy with interferon beta was permitted. Hepatotoxicity of this drug is known and may account partially for the elevation of liver enzymes reported in this study. Our trial was also safe regarding other organ-specific side effects and participants reported good overall tolerability of EGCG.

Immunologic analyses revealed no impact of EGCG on T-cell responses to GA except when applying very high doses of GA, suggesting that the study medication did not counteract the beneficial effects of GA.

In our study cohort, all patients were under stable GA treatment before administering EGCG, and only about half of the participants had suffered from a relapse during 12 months before study inclusion. This is in strong contrast to recent studies on disease-modifying drugs in MS,^30,31^ which report a mean of 1.4 relapses in the previous 12 months, and demonstrates the notable stability of our study population. It is composed of many patients with a more benign course of MS who had a median EDSS of 2.0 at study entry after 8–9 years of disease, making it even more difficult to observe a therapeutic effect in such patients.

As both study groups (GA + EGCG and GA + placebo) also hardly suffered from disease activity during the trial, the absence of disease dynamics made it impossible to detect an effect of the intervention.

Overestimation of the treatment effect calculating the sample size might also account for the difficulties in demonstrating mild additive or synergistic effects of EGCG.

With the given data, the power is only 7% in the ITT population to detect the difference of percentages of patients without new T2w lesions between verum and placebo at the end of the study. This figure is very revealing: the small difference of percentages of patients without new T2w lesions between treatment groups in our cohort (31.9% vs 39.1%) can only be detected with 719 patients per group, assuming a power of 80% and a type 1 error (α) of 5% (two-sided).

These numbers seem very high for a clinical trial, but they are in the order of size of the phase III studies of the substances teriflunomide (TEMSO n = 1,088, TOWER n = 1,169)^30,32^ and dimethyl fumarate (DEFINE n = 1,237, CONFIRM n = 1,430),^30,33^ which are now approved for the treatment of MS. The power considerations keep open the possibility of exploring, with an appropriate study design, whether the disease course of MS (at least in terms of T2 lesion load) can be influenced by EGCG. Although the subgroup analyses only showed a statistical trend because of the small number of cases in the subgroups, it could be speculated with caution that patients with MS without relapse activity and a low cerebral lesion load could benefit from EGCG treatment.

Despite experimental evidence of anti-inflammatory and neuroprotective properties of EGCG,^9^ in the human setting, its neuroprotective capacities may outweigh. There is growing evidence from several case-controlled and cohort studies in North America, Europe, and Asia that consumption of tea lowers the risk of neurodegenerative disease like Alzheimer and Parkinson disease.^34^ However, a recently published phase III controlled clinical trial in multiple system atrophy could not reveal an association with clinically relevant disease modification compared with placebo,^35^ despite—also in this case—promising basic science and animal experimental data.^36^ Also the evaluation of PBVC—a marker for brain atrophy—in our study could not prove an effect of EGCG on neurodegeneration within 18 months. Even with a significant extension of the study period to 36 months, no relevant effect of EGCG on the atrophy rate could be demonstrated in progressive MS.^37^

Recent studies reported beneficial effects of orally applied EGCG on cognitive functions in combination with cognitive training in patients with Down syndrome and fragile X syndrome.^38,39^ In our study, we assessed for the screening of cognitive function the PASAT testing calculation ability, auditory information processing speed, and flexibility. We could not reveal a positive effect of EGCG on this secondary end point. Though, our progressive MS study has provided suggestion that EGCG may have a positive effect on the test performance in PASAT.^37^ As cognitive decline in MS is an overwhelming and up to now unsolved problem, the effect of EGCG on improvement of cognitive function in MS should be investigated in a more sophisticated approach.

EGCG at a dose of up to 800 mg daily was safe and well tolerated in patients with RRMS when given as add-on to GA over 18 months. However, no effect on MRI or clinical measures of disease activity could be demonstrated. Possible explanations include an underestimation of effect size in sample size calculation and insufficient EGCG dosage. Given that recent studies reported beneficial effects on cognitive functions, further investigation of EGCG in MS focused on these aspects may be warranted. Future studies should use optimized dose regimens or newer formulations of EGCG that increase bioavailability and offer an improved safety profile, in particular with regard to liver toxicity.

## Glossary

AE: adverse event
CEL: contrast-enhancing lesion
EAE: experimental autoimmune encephalomyelitis
ECCG: epigallocatechin-3-gallate
EDSS: Expanded Disability Status Scale
GA: glatiramer acetate
ITT: intention to treat
MSFC: MS Functional Composite
NK: natural killer
PASAT: Paced Auditory Serial Addition Test
PBMC: peripheral blood mononuclear cell
PBVC: percent brain volume change
PP: per protocol
RRMS: relapsing-remitting MS
SAE: serious adverse event
